# Evaluation of Functional Recovery Following Thrombectomy in Patients With Large Vessel Occlusion and Prestroke Disability

**DOI:** 10.1001/jamanetworkopen.2022.27139

**Published:** 2022-08-16

**Authors:** Maximilian I. Sprügel, Jochen A. Sembill, Svenja Kremer, Stefan T. Gerner, Michael Knott, Stefan Hock, Tobias Engelhorn, Arnd Dörfler, Hagen B. Huttner, Stefan Schwab

**Affiliations:** 1 Department of Neurology, Friedrich-Alexander-Universität Erlangen-Nürnberg, Erlangen, Germany; 2 Department of Neuroradiology, Friedrich-Alexander-Universität Erlangen-Nürnberg, Erlangen, Germany

## Abstract

**Importance:**

It is uncertain whether thrombectomy is associated with benefits in patients with prestroke disability.

**Objective:**

To evaluate the use of thrombectomy for patients with large vessel occlusion and prestroke disability.

**Design, Setting, and Participants:**

This cohort study included patients with large vessel occlusion stroke and prestroke disability (modified Rankin Scale score, 3 or 4) admitted to a single tertiary care center between January 1, 2006, and June 30, 2019 (controls: 2006-2015; thrombectomy: 2015-2019). Follow-up was conducted at 90 days. Data analysis was performed from November 1 to December 31, 2021.

**Exposures:**

Use of thrombectomy vs no thrombectomy.

**Main Outcomes and Measures:**

The primary outcome was functional recovery at 90 days defined as clinical recovery to the functional status before stroke onset. Secondary outcomes included functional dependency, mortality, early neurologic improvement, and recanalization.

**Results:**

Among 205 patients (149 women [72.7%]; median age, 82 years [IQR, 75-87 years]), 102 individuals (49.8%) received thrombectomy and 103 (50.2%) were controls. Thrombectomy was significantly associated with functional recovery (thrombectomy, 20 [19.6%]; controls, 8 [7.8%]; adjusted difference, 9.4%; 95% CI, 2.2% to 16.7%; *P* = .005). Secondary outcomes showed differences in mortality, early neurologic improvement, and recanalization in favor of thrombectomy treatment. The rate of functional dependency did not differ significantly between the 2 groups (adjusted difference, 8.9%; 95% CI, −2.5% to 20.2%; *P* = .13). The rate of functional recovery after thrombectomy was 44.0% for patients with early neurologic improvement, 29.4% for patients with small infarct volume (<50 mL), and 7.0% for patients with neither of these parameters.

**Conclusions and Relevance:**

Findings of this study suggest that selected patients with prestroke disability may benefit from thrombectomy. However, the thrombectomy-associated increase in functional recovery was small. Therefore, routine use of thrombectomy may not be beneficial among patients with a large ischemic core and infarct volumes less than 50 mL may be necessary to obtain functional recovery. Patients with higher chances of functional recovery are also at an increased risk of survival with substantial disability, indicating potential harms from the intervention; further studies are needed.

## Key Points

**Question:**

Is thrombectomy associated with changes in outcomes in patients with prestroke disability?

**Findings:**

In this cohort study of 205 patients with prestroke disability, thrombectomy was associated with an adjusted increase in functional recovery by 9.4%. Thrombectomy also was associated with reduced mortality.

**Meaning:**

The findings of this study suggest that thrombectomy should be performed only in carefully selected patients with prestroke disability.

## Teaser

This cohort study examines functional outcomes following the use of thrombectomy in patients who have functional disability before large vessel occlusion stroke.

## Introduction

Stroke is a leading cause of disability worldwide.^1,2^ Acute treatment strategies have greatly advanced in recent years, with thrombectomy and intravenous thrombolysis leading to rapid reperfusion and reduced disability.^3^

Approximately 20% of patients with stroke in a clinical practice setting have impaired functional status before the presenting stroke, but these patients were not included in randomized clinical trials evaluating treatment strategies.^4,5,6^ Observational cohort studies suggest that the intervention was safe in patients with prestroke disability and thrombectomy might be a factor in maintaining the premorbid functional status.^7,8^ However, these studies lacked a control group of patients not receiving thrombectomy, and intervention-associated differences in outcomes therefore could not be determined.^7,8^ Furthermore, thrombectomy might reduce mortality and even increase the rate of stroke survival with functional dependency.^9,10^

The purpose of the present study was to investigate the association of thrombectomy with (1) functional recovery, (2) functional dependency, (3) mortality, (4) early neurologic improvement, (5) recanalization, (6) infarct volume, and (7) secondary safety outcomes among patients with prestroke disability.

## Methods

Consecutive patients with ischemic stroke admitted to the University Hospital Erlangen, Germany, between January 1, 2006, and June 30, 2019, were recruited in a longitudinal cohort study, the Stroke Research Consortium in Northern Bavaria.^11^ In addition to patients receiving stroke treatment (thrombolysis and thrombectomy), this cohort study included patients not receiving such interventions to characterize treatment-associated differences in outcomes between a treatment and control group. Included patients had ischemic stroke with large vessel occlusion not receiving thrombectomy or thrombolysis; ischemic stroke with large vessel occlusion receiving thrombectomy with or without thrombolysis; or ischemic stroke receiving thrombolysis. Analyses were performed among patients with large vessel occlusion not receiving thrombectomy or thrombolysis (control group: patients admitted between January 1, 2006, and December 31, 2015, because thrombectomy was not routinely performed during this period) and patients with large vessel occlusion who had prestroke disability receiving thrombectomy with or without thrombolysis (intervention group: patients admitted between January 1, 2015, and June 30, 2019, because thrombectomy was routinely performed during this period; in 2015, 5 randomized clinical trials showed efficacy of thrombectomy and changed standard of care; thus, 2015 represents a transition period and was included in both groups).^6^ The study was reviewed and approved by the Friedrich-Alexander-University Erlangen-Nuremberg, Germany institutional review board. Patients or their legal representatives provided informed consent unless waived by the review board (>50% of patients died within 90 days and a substantial proportion died during their hospital stay). This study followed the Strengthening the Reporting of Observational Studies in Epidemiology (STROBE) reporting guideline.

### Definitions and Data Acquisition

Data on demographic characteristics (age and sex), comorbidities, status on hospital admission, and intrahospital parameters were obtained, as previously published.^12,13,14^ Prestroke functional status was assessed at hospital admission using the modified Rankin Scale (mRS) based on information by patients, relatives, medical records, and if applicable, caregivers and referring physicians.^15^ Prestroke disability was defined as a score on the mRS of 3 or 4.^7,8^ Recanalization was assessed by color-coded duplex sonography of extracranial arteries and color-coded duplex or Doppler sonography of intracranial arteries, or computed tomography (CT) or magnetic resonance imaging (MRI) angiography of extracranial and intracranial arteries.^16,17^ Infarct volumes were assessed by CT perfusion and MRI diffusion and perfusion imaging, using the automated RAPID software (iSchemaView Inc); details on vascular imaging, infarct volume assessment, and control imaging are presented in the eMethods in the Supplement.^10,18,19^ Trained raters assessed follow-up at 90 days by telephone interview, outpatient visits, or medical reports.^12^

### Outcomes

The primary outcome was functional recovery at 90 days, defined as clinical recovery to the functional status before stroke onset (ie, prestroke score on the mRS achieved at 90 days).^20^ Secondary outcomes comprised (1) functional dependency at 90 days, defined as clinical worsening of the functional status before stroke onset among survivors (ie, prestroke score on the mRS not achieved and alive at 90 days); (2) mortality at 90 days; (3) early neurologic improvement, defined as a decrease in the National Institutes of Health Stroke Scale (NIHSS) score of 10 points or more from baseline or an NIHSS score of 0 or 1 on day 7 after hospital admission or at discharge, whichever occurred first^18^; (4) recanalization assessed on vascular follow-up imaging or based on clinical and radiologic findings; (5) grade 2b indicating reperfusion of 50% to 90% or grade 3 indicating complete resolution of the affected territory on the modified Treatment in Cerebral Ischemia scale after thrombectomy^7,10^; and (6) median infarct volume at day 2 assessed on follow-up imaging. Secondary safety outcomes were (1) early neurologic deterioration, defined as an increase in the NIHSS score of 4 or more points within 5 days after the stroke that was not attributed to intracranial hemorrhage or malignant cerebral edema^18^; (2) symptomatic intracranial hemorrhage, defined as brain hemorrhage associated with an increase of at least 4 points on the NIHSS^10^; and (3) parenchymal hematoma type 2, defined as a hematoma occupying 30% or more of the infarcted tissue with obvious mass effect.^21^ Size of the infarct area was estimated using the Alberta Stroke Program Early CT Score (ASPECTS).

### Statistical Analyses

All statistical analyses were performed using IBM SPSS Statistics, version 24.0 (SPSS Institute Inc) from November 1 to December 31, 2021. Two-sided statistical tests were applied with a significance level of *P* = .05. Categorical variables were evaluated using the χ^2^ test or Fisher exact test where appropriate and are reported as total numbers and percentages. Ordinal and continuous variables were evaluated using the Mann-Whitney test and are reported as medians (IQRs). Multivariable regression models were applied to analyze the association between thrombectomy and outcomes with adjustment for parameters that showed differences in intergroup comparison and are relevant for outcomes among patients not receiving thrombectomy (parameters not relevant for outcomes among patients not receiving thrombectomy were not included, eg, time from first observation of symptoms to admission), as well as for parameters associated with functional outcome. Adjusted differences were estimated using a general linear model with adjustment for the same parameters. Subgroup analyses were performed for functional recovery (primary outcome) and functional dependency (secondary outcome) among different subgroups (eMethods in the Supplement).^9,10,18,22,23,24^ Sensitivity analyses were conducted for the primary outcome (eTable in the Supplement).

## Results

### Patient Characteristics

Of 262 patients reviewed, the study sample comprised 205 patients with large vessel occlusion stroke and prestroke disability; 149 were women (72.7%), 56 were men (27.3%), and the median age was 82 (IQR, 75-87) years. Fifty-seven of 262 patients were excluded from the analyses because of posterior circulation, anterior cerebral artery or third segment of middle cerebral artery occlusion, or missing data (eFigure 1 in the Supplement). Thrombectomy was performed in 102 of 205 patients (49.8%). Data on volume of ischemic core and volume of perfusion lesion were available on 77 patients (75.5%) in the thrombectomy group and 70 of 103 patients (68.0%) in the control group in whom perfusion imaging was performed on hospital admission. Data on recanalization were obtained by sonography in 176 patients and CT or MRI angiography in 8 patients at a median of 23.9 (IQR, 15.6-39.8) hours after initial imaging; in 21 patients, recanalization was assessed by clinical and radiologic findings. Median infarct volume was assessed in 193 patients, with follow-up imaging performed at a median of 23.6 (IQR, 19.1-30.7) hours after hospital admission; in 22 patients, infarct volume was assessed on initial imaging.

There were no significant differences in age, sex, prestroke functional status, and comorbidities between the 2 groups (Table 1). Volume of the ischemic core was smaller in the thrombectomy group vs the control group (8.0 [IQR, 0.0-43.5] mL vs 50.5 [28.8-124.5] mL; *P* < .001), the Alberta Stroke Program Early CT Score (ASPECTS) was higher (8 [IQR, 7-10] vs 7 [IQR, 5-8]; *P* < .001), and the volume of the perfusion lesion was numerically but not significantly smaller (123.0 [IQR, 94.5-183.5] mL vs 153.0 [IQR, 99.3-206.0] mL; *P* = .12). The rate of internal carotid artery occlusion was not significantly lower in the thrombectomy vs control group (31 of 102 [30.4%] vs 44 of 103 [42.7%] patients; *P* = .07) and the rate of first segment of middle cerebral artery occlusion (57 [55.9%] vs 46 [44.7%] patients; *P* = .11) was not significantly higher. There also was no significant difference between the thrombectomy and control groups regarding the time from last known well to admission (7.5 [IQR, 4.0-13.6] h vs 10.9 [IQR, 6.7-13.7] h; *P* = .10), but the difference between the groups in time from first observation of symptoms to admission was significant (1.9 [IQR, 1.0-3.2] h vs 3.7 [IQR, 1.8-10.4] h; *P* < .001). Treatment with intravenous alteplase was performed in 61 of 102 patients (59.8%) in the thrombectomy group vs none in the control group. Outcomes were evaluated using multivariable regression analysis adjusted for occlusion site (internal carotid artery vs middle cerebral artery; relevant parameters showing differences in intergroup comparison), age, and NIHSS score (parameters associated with functional outcome).

**Table 1.** Baseline Characteristics

Characteristic	No. (%)		*P* value
	Thrombectomy group (n = 102)	Control group (n = 103)^a^	
Age, median (IQR), y	82 (77-87)	81 (72-87)	.63
Sex			
Female	74 (72.5)	75 (72.8)	.97
Male	28 (27.5)	28 (27.2)	.97
Prestroke functional status			
Score of 3 on mRS	82 (80.4)	80 (77.7)	.63
Score of 4 on mRS	20 (19.6)	23 (22.3)	.63
Atrial fibrillation	60 (58.8)	55 (53.4)	.43
Anticoagulation therapy	22 (21.6)	16 (15.5)	.27
Diabetes	41 (40.2)	38 (36.9)	.63
Hypertension	87 (85.3)	83 (80.6)	.37
Previous ischemic stroke or TIA	31 (30.4)	37 (35.9)	.40
NIHSS score, median (IQR)	18 (14-21)	18 (12-22)	.82
Treatment with intravenous alteplase	61 (59.8)	0	<.001
Imaging characteristics			
CT perfusion imaging	70 (68.6)	34 (33.0)	<.001
MRI perfusion imaging	7 (6.9)	36 (35.0)	<.001
Volume of ischemic core, median (IQR), mL^b^	8.0 (0.0-43.5)	50.5 (28.8-124.5)	<.001
Volume of perfusion lesion, median (IQR), mL^b^	123.0 (94.5-183.5)	153.0 (99.3-206.0)	.12
Occlusion site			
Internal carotid artery	31 (30.4)	44 (42.7)	.07
First segment of middle cerebral artery	57 (55.9)	46 (44.7)	.11
Second segment of middle cerebral artery	14 (13.7)	13 (12.6)	.82
ASPECTS on baseline imaging, median (IQR)	8 (7-10)	7 (5-8)	<.001
Type of stroke onset			
Unwitnessed stroke	51 (50.0)	62 (60.2)	.14
Witnessed stroke	51 (50.0)	41 (39.8)	.14
Process measures, median (IQR), h			
Time from last known well to admission	7.5 (4.0-13.6)	10.9 (6.7-13.7)	.10
Time from first observation of symptoms to admission	1.9 (1.0-3.2)	3.7 (1.8-10.4)	<.001
Time from imaging to femoral puncture	0.9 (0.4-1.3)	NA	NA
Time from femoral puncture to reperfusion	0.8 (0.6-1.2)	NA	NA

Abbreviations: ASPECTS, Alberta Stroke Program Early CT Score; CT, computed tomography; IQR, interquartile range; MRI, magnetic resonance imaging; mRS, modified Rankin scale; NA, not applicable; NIHSS, National Institutes of Health Stroke Scale; TIA, transient ischemic attack.

^a^ The control group represents patients with large vessel occlusion stroke not receiving thrombectomy or thrombolysis, admitted between January 1, 2006, and December 31, 2015.

^b^ Data were available for 77 of 102 patients (75.5%) in the thrombectomy group and 70 of 103 patients (68.0%) in the control group in whom perfusion imaging was performed on hospital admission.

### Outcomes

Regarding the primary outcome, thrombectomy was associated with functional recovery at 90 days (thrombectomy, 20 [19.6%] vs controls, 8 [7.8%]; adjusted difference, 9.4%; 95% CI, 2.2%-16.7%; adjusted odds ratio [aOR], 4.33; 95% CI, 1.55-12.10; *P* = .005) (Table 2). Regarding secondary outcomes, the rate of functional dependency at 90 days did not differ significantly between the 2 groups (thrombectomy, 28 [27.5%] vs controls, 19 [18.4%]; adjusted difference, 8.9%; 95% CI, −2.5% to 20.2%; aOR, 1.70; 95% CI, 0.86-3.33; *P* = .13). There were significant differences between the thrombectomy and control groups in mortality at 90 days (thrombectomy, 54 [52.9%] vs 76 [73.8%]; adjusted difference, −24.4%; 95% CI, −38.1% to −10.6%; aOR, 0.32; 95% CI, 0.17-0.62; *P* < .001), early neurologic improvement (25 [24.5%] vs 4 [3.9%]; adjusted difference, 18.1%; 95% CI, 8.9%-27.3%; aOR, 7.54; 95% CI, 2.50-22.75; *P* < .001), recanalization (92 [90.2%] vs 17 [16.5%]; adjusted difference, 77.3%; 95% CI, 67.9%-86.6%; aOR, 62.93; 95% CI, 24.33-162.76; *P* < .001), and infarct volume at day 2 (median, 40.5 [IQR, 4.0-108.4] mL vs 133.0 [IQR, 81.5-186.0] mL; *P* < .001 [adjusted difference and aOR not determined]). Reperfusion after the intervention (grade 2b or 3 on the modified Treatment in Cerebral Ischemia scale) was achieved in 89 of 102 patients (87.3%) in the thrombectomy group.

**Table 2.** Primary and Secondary Outcomes

Outcome	No. (%)		Adjusted difference, % (95% CI)^a^	Adjusted odds ratio (95% CI)^b^	*P* value
	Thrombectomy group (n = 102)	Control group (n = 103)			
Primary					
Functional recovery at 90 d	20 (19.6)	8 (7.8)	9.4 (2.2 to 16.7)	4.33 (1.55-12.10)	.005
Secondary					
Functional dependency at 90 d^c^	28 (27.5)	19 (18.4)	8.9 (−2.5 to 20.2)	1.70 (0.86-3.33)	.13
Mortality at 90 d	54 (52.9)	76 (73.8)	−24.4 (−38.1 to −10.6)	0.32 (0.17-0.62)	<.001
Early neurologic improvement^d^	25 (24.5)	4 (3.9)	18.1 (8.9 to 27.3)	7.54 (2.50-22.75)	<.001
Recanalization^e^	92 (90.2)	17 (16.5)	77.3 (67.9 to 86.6)	62.93 (24.33-162.76)	<.001
Grade of 2b or 3 on mTICI scale^f^	89 (87.3)	NA	NA	NA	NA
Infarct volume at day 2, median (IQR), mL^g^	40.5 (4.0-108.4)	133.0 (81.5-186.0)	NA	NA	<.001
Secondary safety					
Early neurologic deterioration^h^	8 (7.8)	19 (18.4)	−5.7 (−13.4 to 2.0)	0.48 (0.18-1.28)	.14
Symptomatic intracranial hemorrhage	6 (5.9)	1 (1.0)	4.9 (−0.0 to 9.9)	6.38 (0.75-53.93)	.09
Parenchymal hematoma type 2	5 (4.9)	1 (1.0)	3.9 (−0.7 to 8.5)	5.26 (0.60-45.82)	.13

Abbreviations: mTICI, modified Treatment in Cerebral Ischemia; NA, not applicable.

^a^ Adjusted differences were estimated using general linear model with adjustment for age, National Institutes of Health Stroke Scale (NIHSS) score, and occlusion site.

^b^ Multivariable regression analyses were adjusted for age, NIHSS score, and occlusion site. Final infarct volume and symptomatic intracranial hemorrhage and parenchymal hematoma type 2 represent unadjusted analyses.

^c^ Defined as worsening of functional status before stroke onset among survivors of stroke at 90 days.

^d^ Defined as decrease in the NIHSS score of 10 points or more from baseline or an NIHSS score of 0 or 1 within 7 days after hospital admission.

^e^ Assessed by sonography in 176 patients and computed tomography or magnetic resonance imaging angiography in 8 patients at a median of 23.9 (IQR, 15.6-39.8) hours after initial imaging. In 21 patients, recanalization was assessed by clinical and radiologic findings.

^f^ Score ranges from 0 to 3, with grade 2b indicating reperfusion of 50% to 90% and grade 3 indicating complete reperfusion of the affected territory.

^g^ Assessed in 193 patients, with follow-up imaging performed at a median of 23.6 (IQR, 19.1-30.7) hours after hospital admission. In 22 patients, infarct volume was assessed on initial imaging.

^h^ Defined as an increase in the NIHSS score of 4 or more points within 5 days after the stroke that was not attributed to intracranial hemorrhage or malignant cerebral edema.

Regarding secondary safety outcomes, the rate of early neurologic deterioration did not differ significantly between the thrombectomy and control groups (8 [7.8%] vs 19 [18.4%]; adjusted difference, −5.7%; 95% CI, −13.4% to 2.0%; aOR, 0.48; 95% CI, 0.18-1.28; *P* = .14). Rates of symptomatic intracranial hemorrhage and parenchymal hematoma type 2 were numerically higher in patients treated with thrombectomy. Rates of procedure-related complications were low (Table 3).

**Table 3.** Thrombectomy-Associated Complications

Complication	Thrombectomy patients, No. (%) (n = 102)
Distal embolization in a different territory	4 (3.9)
Intramural arterial dissection	2 (2.0)
Arterial perforation	2 (2.0)
Access-site complications leading to intervention	2 (2.0)

### Subgroup Analyses

**Figure 1.**
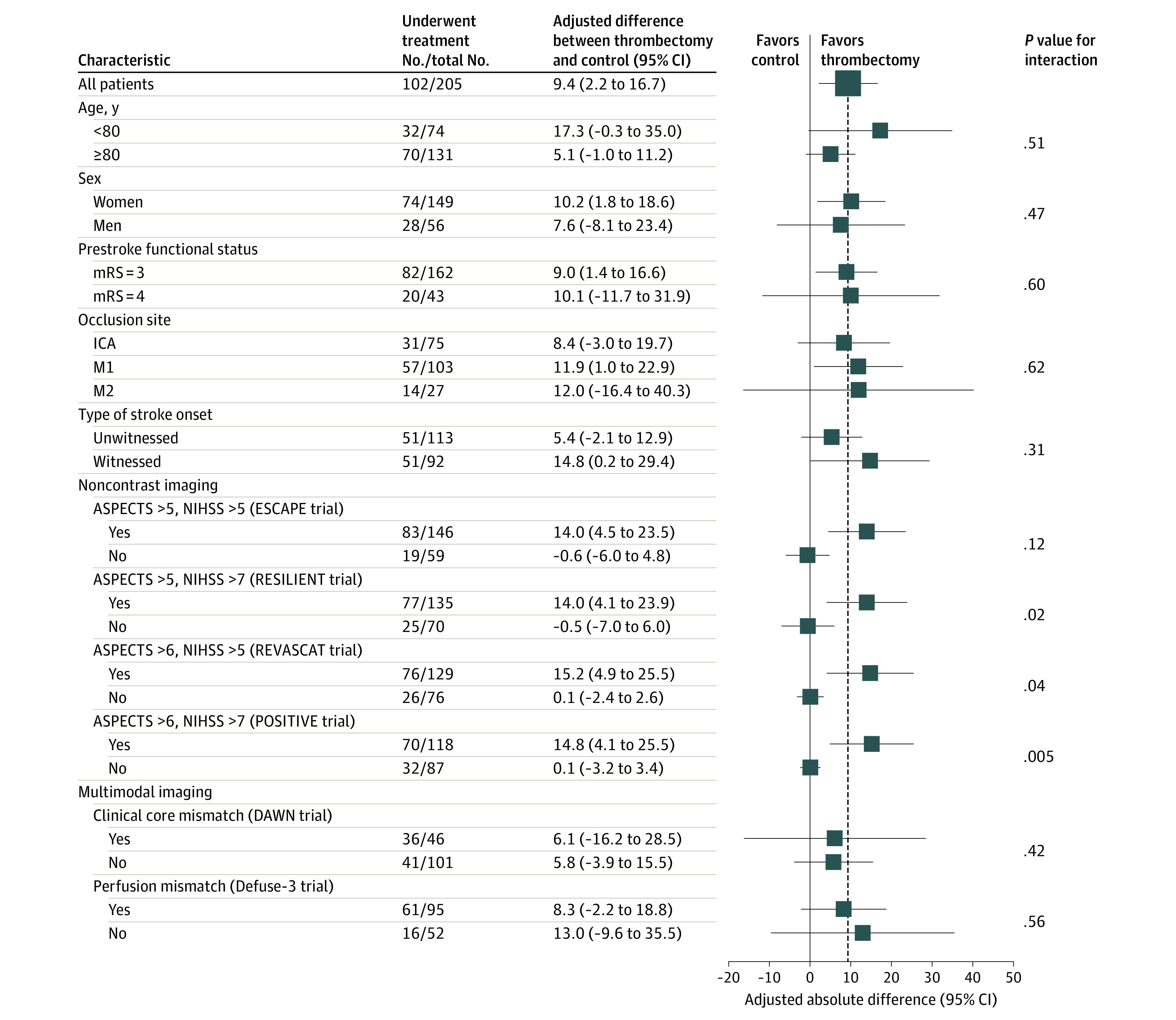
Subgroup Analysis of the Primary Outcome Subgroup analyses were performed using general linear model with adjustment for age, National Institutes of Health Stroke Scale (NIHSS) score and occlusion site to estimate adjusted differences. Alberta Stroke Program Early CT Score (ASPECTS) and NIHSS definitions were based on simplified trial inclusion and exclusion criteria. ICA indicates internal carotid artery; M1, first segment of middle cerebral artery; M2, second segment of middle cerebral artery; and mRS, modified Rankin Scale.

Regarding functional recovery, there were interactions among subgroups according to size of the infarct area and clinical deficits (Figure 1). Patients with moderate to severe neurologic deficits and small to moderate ischemic core had larger adjusted differences of functional recovery between the thrombectomy and control groups. Patients with ASPECTS greater than 5 and NIHSS scores greater than 7 (based on simplified criteria of the RESILIENT trial^22^) had an adjusted difference of 14.0% (95% CI, 4.1%-23.9%) for functional recovery compared with −0.5% (95% CI, −7.0% to 6.0%) for patients with large ischemic core or minor clinical deficits (*P* = .02 for interaction). There was no statistically significant heterogeneity among subgroups characterized by multimodal imaging, but patient numbers were limited. Regarding functional dependency, there were interactions among similar subgroups (eFigure 2 in the Supplement). Patients with ASPECTS greater than 5 and NIHSS score greater than 7 had an adjusted difference of 11.7% (95% CI, −3.3% to 26.8%) for functional dependency compared with −3.6% (95% CI, −16.6% to 9.4%) for patients with large ischemic core or minor clinical deficits (*P* = .02 for interaction).

### Sensitivity and Exploratory Analyses

**Figure 2.**
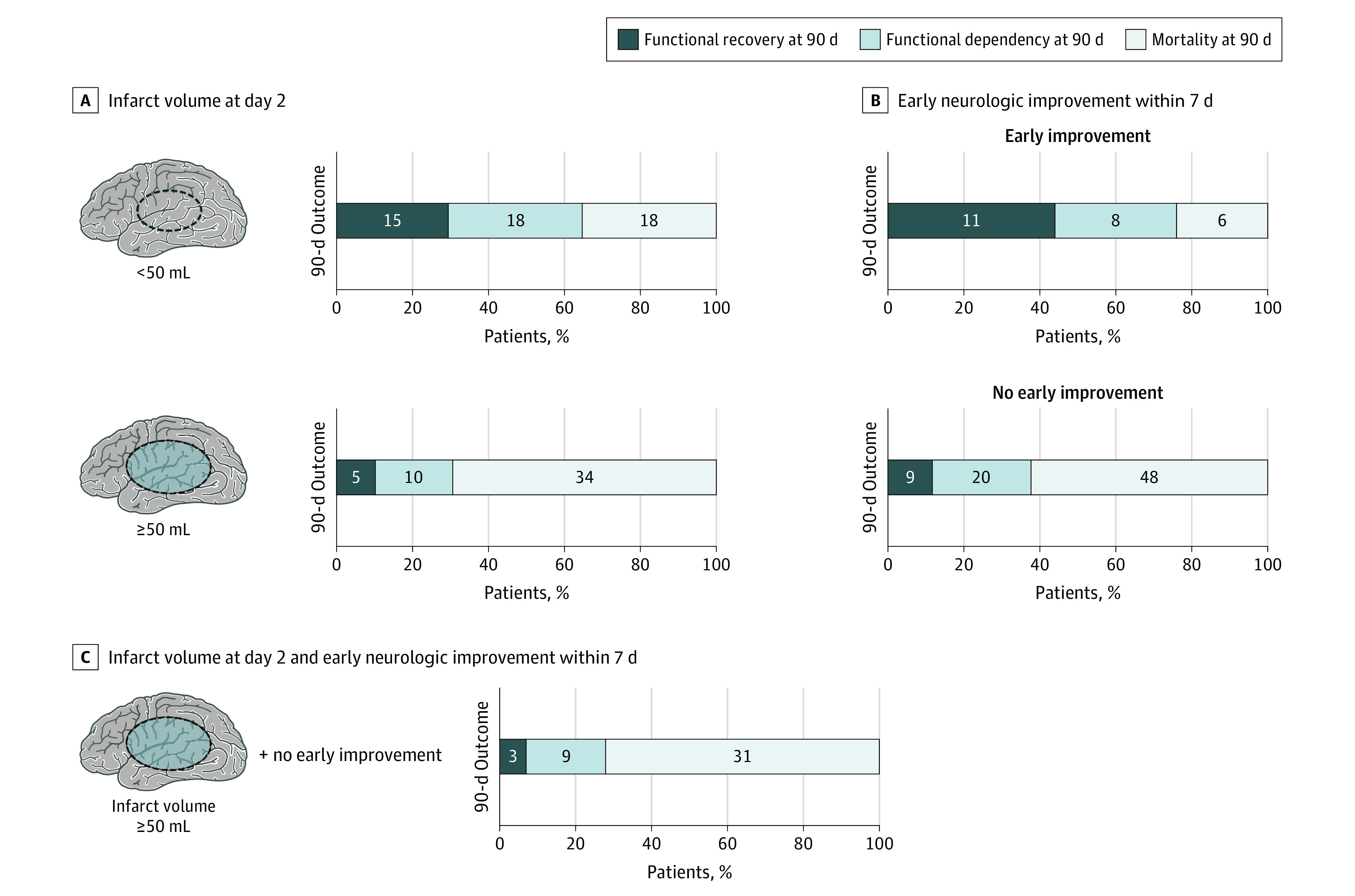
Exploratory Analysis of Clinical Outcomes According to Infarct Volume and Early Neurologic Improvement After Thrombectomy Functional recovery, functional dependency, and mortality at 90 days were evaluated according to infarct volume at day 2 (A), early neurologic improvement within 7 days (B), and for the subgroup of patients with infarct volume greater than or equal to 50 mL and without early neurologic improvement (C). Early neurologic improvement was defined as a decrease in the National Institutes of Health Stroke Scale (NIHSS) score of 10 points or more from baseline or an NIHSS score of 0 or 1 within 7 days after hospital admission.

Sensitivity analyses according to in-hospital treatment, rehabilitation, and imaging parameters yielded similar results (eTable in the Supplement). Because size of the infarct area and clinical deficits interact with the thrombectomy-associated increase in functional recovery, we evaluated clinical outcomes after thrombectomy according to infarct volume at day 2 and early neurologic improvement until day 7 (Figure 2). Functional recovery was more frequent among patients with small infarct volume (<50 mL, 15 [29.4%] vs ≥50 mL, 5 [10.2%]; *P* = .02) (Figure 2A) and among patients with early neurologic improvement within 7 days (11 [44.0%] vs 9 [11.7%]; *P* < .001) (Figure 2B). Less than 7.0% of patients with large infarct volume (≥50 mL) and without early neurologic improvement achieved functional recovery after thrombectomy (Figure 2C).

## Discussion

To our knowledge, there have been no randomized clinical trials evaluating thrombectomy in patients with prestroke disability and, to our knowledge, this study represents the first comprehensive observational cohort analysis including a control group of patients not receiving thrombectomy. We found a small thrombectomy-associated increase in functional recovery, but increased survival may be at the expense of substantial disability, thus presenting the challenging issue of whether patients with prestroke disability benefit from thrombectomy.

The risk of survival with substantial disability has been discussed for decompressive craniectomy in older patients with space-occupying infarction.^25,26^ Current guidelines suggest that the intervention should be considered to reduce the risk of death in patients aged 61 years or older.^25,26^ However, guidelines of the European Stroke Organisation state that “surgery should only be done after a shared decision process including a careful discussion with the patient or his/her representatives about the risk of survival with substantial disability.”^26^ Similar considerations apply to thrombectomy in patients with prestroke disability, and treatment decisions must be in line with patients’ preferences.^27^

Regarding potential benefits of the intervention, our study findings suggest that thrombectomy with or without intravenous thrombolysis is associated with higher functional recovery rates in patients with prestroke disability. However, outcome rates were low and less than 20% of patients in the intervention group achieved functional recovery. Previous studies reported between 21% and 38% of patients with prestroke disability maintained their premorbid functional status after thrombectomy.^7,8,20^ Because these studies lacked a control group of patients not receiving thrombectomy, intervention-associated differences in outcomes could not be determined and substantial selection bias may be suspected. In contrast, thrombectomy was routinely applied in patients with stroke rather than independent from the functional status before stroke onset at our institution, which is underlined by similar patient characteristics between both groups in this study. Furthermore, the presence of a control group allowed statistical adjustment for relevant parameters, after which thrombectomy was associated with an adjusted increase in functional recovery by 9.4%. Treating physicians may consider this finding and not overestimate any benefits of the intervention in patients with prestroke disability because treatment effects of thrombectomy are up to 4 times higher in patients without prestroke disability.^10,18,28,29^

The findings of our study suggest that patients with large ischemic core (ASPECTS ≤5) do not benefit from thrombectomy. In contrast, a recently published randomized clinical trial showed that thrombectomy improves functional outcomes in patients with large ischemic region (ASPECTS 3-5).^30^ However, this trial included only patients with a score of 0 or 1 on the mRS before stroke onset and our study findings underline that these results do not apply to patients with prestroke disability. This difference could be explained by a lower rehabilitation potential in patients with prestroke disability, because rates of functional recovery were very low among patients with large infarct volume and those who did not show early neurologic improvement. The rate of functional recovery was substantially higher among patients with small infarct volume and the objective of thrombectomy should be infarct volumes of less than 50 mL to obtain reasonable chances of functional recovery in patients with prestroke disability.

Regarding potential harms of the intervention, our study findings suggest that thrombectomy might increase the risk of survival with substantial disability in patients with prestroke disability. Thrombectomy reduces infarct volume, mass effect, and herniation and was reported to decrease mortality rates.^9,10,31^ This could result in higher rates of individuals with prestroke disability who survive stroke but have functional dependency. In the overall cohort of this study, thrombectomy was associated with reduced mortality and with an adjusted nonsignificant increase in functional dependency by 8.9%. Patients with moderate to severe neurologic deficits and small to moderate ischemic core had higher chances of functional recovery but were also at an increased risk of survival with substantial disability. These potential harms from the thrombectomy intervention may be considered by treating physicians and addressed by further studies.

Poststroke rehabilitation is recommended by international guidelines.^32,33^ Rehabilitation programs commonly last for several months and improve function and patients’ quality of life.^33,34^ However, patients with severe stroke have relevant rehabilitation challenges, longer length of stay, and poorer functional outcomes, and recovery efforts are substantially higher for patients and their relatives and other caregivers.^32,35^ These obstacles may be considerably greater for patients with prestroke disability, and less than 6% of individuals who survived stroke retrospectively agreed to neurocritical care if they were in a functionally dependent status 1 year after the stroke event.^36^ In the present study, we identified subgroups of patients with prestroke disability who have relevant rehabilitation potential after thrombectomy with or without intravenous thrombolysis. Functional recovery was noted in 29.4% of patients in whom infarct volumes were less than 50 mL and in 44.0% of those with early neurologic improvement; it may be useful to recommend rehabilitation programs for these patients. However, functional improvement was noted in less than 7.0% of patients with large infarct volume (≥50 mL) and without early neurologic improvement, and the rate of survival with substantial disability was almost 3 times higher. Awareness of these findings may aid in the decision-making process with patients and their relatives. Other appropriate factors for discussion are available treatment options, including palliative care, and patients’ preferences.^37,38,39^ Thrombectomy therapy substantially improved management of treatment in patients with stroke, but it will not improve the individual outcome of every patient with prestroke disability and potential harms associated with false hope, and survival with substantial disability may be detrimental.^37,38,39^

Furthermore, costs of stroke treatment and postacute care pose challenges for health care systems (acute hospitalization and thrombectomy: approximately $45 000 per patient; rehabilitation care and home health care/nursing facility: approximately $81 000 for 1 patient with functional dependency; costs vary among different health care systems).^40,41^ Changes in life expectancy and demographic characteristics will increase the proportion of patients with prestroke disability, and further research is needed to evaluate the benefits and harms of thrombectomy among this patient subgroup.^42^

### Limitations

This study has limitations. Residual confounding is a major limitation. More aggressive stroke treatment over the past few years could result in increased rates of functional recovery in the thrombectomy group and overstate treatment benefits. In addition, only patients in the thrombectomy group received intravenous thrombolysis, which could result in overstated thrombectomy-associated benefits.^43^ However, this confounding underlines the study’s conclusion that benefits of thrombectomy should not be overestimated in patients with prestroke disability. Furthermore, single-center design and retrospective analysis may limit generalizability. Most patients were women and the median age was high. More patients in the thrombectomy group were transferred from primary and secondary stroke centers for the intervention. The sample size was small, limiting adjustment for potential confounders and assessment of associations. ASPECTS values were assessed on both CT and MRI on admission. Patient numbers for subgroup analyses were limited, notably for those with available multimodal imaging data.

## Conclusions

In this study, thrombectomy was associated with improved functional recovery in patients with prestroke disability. However, intervention-associated benefits were small and thrombectomy might increase the risk of survival with substantial disability. Routine use of thrombectomy may not be beneficial among patients with large ischemic core (ASPECTS ≤5) or minor neurologic deficits (NIHSS score ≤5). The appropriate objective of thrombectomy may be infarct volumes of less than 50 mL to obtain reasonable chances of functional recovery. Discussion of rehabilitation programs for patients with small infarct volumes or early neurologic improvement may be useful. In addition, the decision-making process may be facilitated if patients with large infarct volume (≥50 mL) and without early neurologic improvement are informed of the potential lack of benefit with thrombectomy, because less than 7.0% of these patients achieved functional recovery in the present study. Further research is needed to assess both benefits and harms of the intervention.

## Notes

Supplement. **eMethods.** Detailed Methods **eFigure 1.** Flow Chart of Study Participants **eFigure 2.** Subgroup Analysis of Functional Dependency (Secondary Outcome) **eTable.** Sensitivity Analyses
