# Supplementary material for: Evaluation of Functional Recovery Following Thrombectomy in Patients With Large Vessel Occlusion and Prestroke Disability
Source: JAMA Netw Open. 2022 Aug 16;5(8):e2227139. doi: 10.1001/jamanetworkopen.2022.27139 (PMC9382438; doi:10.1001/jamanetworkopen.2022.27139)
Supplement: Supplement. — eMethods. Detailed Methods eFigure 1. Flow Chart of Study Participants eFigure 2. Subgroup Analysis of Functional Dependency (Secondary Outcome) eTable. Sensitivity Analyses [file jamanetwopen-e2227139-s001.pdf]

Supplementary Online Content

Sprügel MI, Sembill JA, Kremer S, et al. Evaluation of functional recovery following thrombectomy in patients with large vessel occlusion and prestroke disability. *JAMA Netw Open*. 2022;5(8):e2227139. doi:10.1001/jamanetworkopen.2022.27139

eMethods. Detailed Methods

eFigure 1. Flow Chart of Study Participants

eFigure 2. Subgroup Analysis of Functional Dependency (Secondary Outcome)

eTable. Sensitivity Analyses

This supplementary material has been provided by the authors to give readers additional information about their work.

© 2022 Sprügel MI et al. *JAMA Network Open.*

eMethods. Detailed Methods

Missing data was below 2.0% except for perfusion imaging parameters on hospital admission (28.3%) and median infarct volume at day 2 (5.9%). Vascular imaging was routinely performed on the day following hospital admission. In those patients without vascular follow-up imaging, assessment of recanalization was based on clinical and radiologic findings (i.e. recanalization: successful reperfusion by EVT and neurological stability [defined as difference between National Institutes of Health Stroke Scale (NIHSS) at 24 hours and after racanalization: -3 to 3]; no recanalization: complete infarction in vascular territory on imaging, or vessel occlusion and no EVT and neurological stability, or persistent vessel occlusion after EVT and neurological stability).

Infarct volumes were assessed by CT perfusion, and MRI diffusion and perfusion imaging using the automated RAPID software. Volume of ischemic core was defined as substantial reduction in cerebral blood flow (CBF<30%) or substantial reduction in tissue diffusion (apparent diffusion coefficient, ADC<620 $*10^{-6}mm^2/s$), respectively, and volume of perfusion lesion as substantial hypoperfusion (time to maximum of the residue function, Tmax >6s) [10, 17]. If automated imaging analysis by the RAPID software was not possible, infarct volumes were manually assessed with equivalent perfusion thresholds. ASPECTS was assessed using the automated RAPID software on non-contrast CT or diffusion-weighted MRI on hospital admission [18]. If automated imaging analysis by the RAPID software was not possible, ASPECTS values were manually assessed.

Control-imaging was routinely performed by non-contrast CT approximately 24 hours after hospital admission to assess intracranial hemorrhage and infarct volume. In some patients without follow-up imaging, assessment of infarct volume was based on clinical and radiologic findings (i.e. complete infarction in vascular territory on initial imaging or persistent vessel occlusion and available perfusion lesion on initial imaging and neurological stability or volume of ischemic core equivalent to volume of perfusion lesion on initial imaging).

Subgroup analyses were performed for functional recovery (primary outcome) and functional dependency (secondary outcome) among the subgroups of patients according to age (<80 years, ≥80 years), sex (women, men), prestroke functional status (prestroke mRS=3, mRS=4), occlusion site (internal carotid artery, first segment and second segment of middle cerebral artery), type of stroke onset (unwitnessed, witnessed), non-contrast imaging (ASPECTS>5 and NIHSS>5 [criteria of Endovascular Treatment for Small Core and Anterior Circulation Proximal Occlusion with Emphasis on Minimizing CT to Recanalization Times, ESCAPE trial[9]]; ASPECTS>5 and NIHSS>7 [Randomization of Endovascular Treatment with Stent-retriever and/or Thromboaspiration versus Best Medical Therapy in Acute Ischemic Stroke due to Large Vessel Occlusion Trial, RESILIENT trial[21]]; ASPECTS>6 and NIHSS>5 [Randomized Trial of Revascularization with Solitaire FR Device versus Best Medical Therapy in the Treatment of Acute Stroke Due to Anterior Circulation Large Vessel Occlusion Presenting within Eight Hours of Symptom Onset, REVASCAT trial[22]]; ASPECTS>6 and NIHSS>7 [Perfusion imaging selection of ischemic stroke patients for endovascular therapy, POSITIVE trial[23]]) and multimodal imaging parameters (clinical core mismatch defined as age≥80years, NIHSS ≥10 and ischemic core <21ml or age <80years, NIHSS ≥10 and ischemic core <31ml or age <80 years, NIHSS ≥20 and ischemic core <51ml [DWI or CTP Assessment with Clinical Mismatch in the Triage of Wake-Up and Late Presenting Strokes Undergoing Neurointervention with Trevo, DAWN trial[17]]; ischemic core <70ml, mismatch ratio ≥1.8, mismatch volume ≥15ml and NIHSS >5 [Endovascular Therapy Following Imaging Evaluation for Ischemic Stroke, DEFUSE 3 trial[10]]).

© 2022 Sprügel MI et al. *JAMA Network Open.*

eFigure 1: Flow chart of study participants

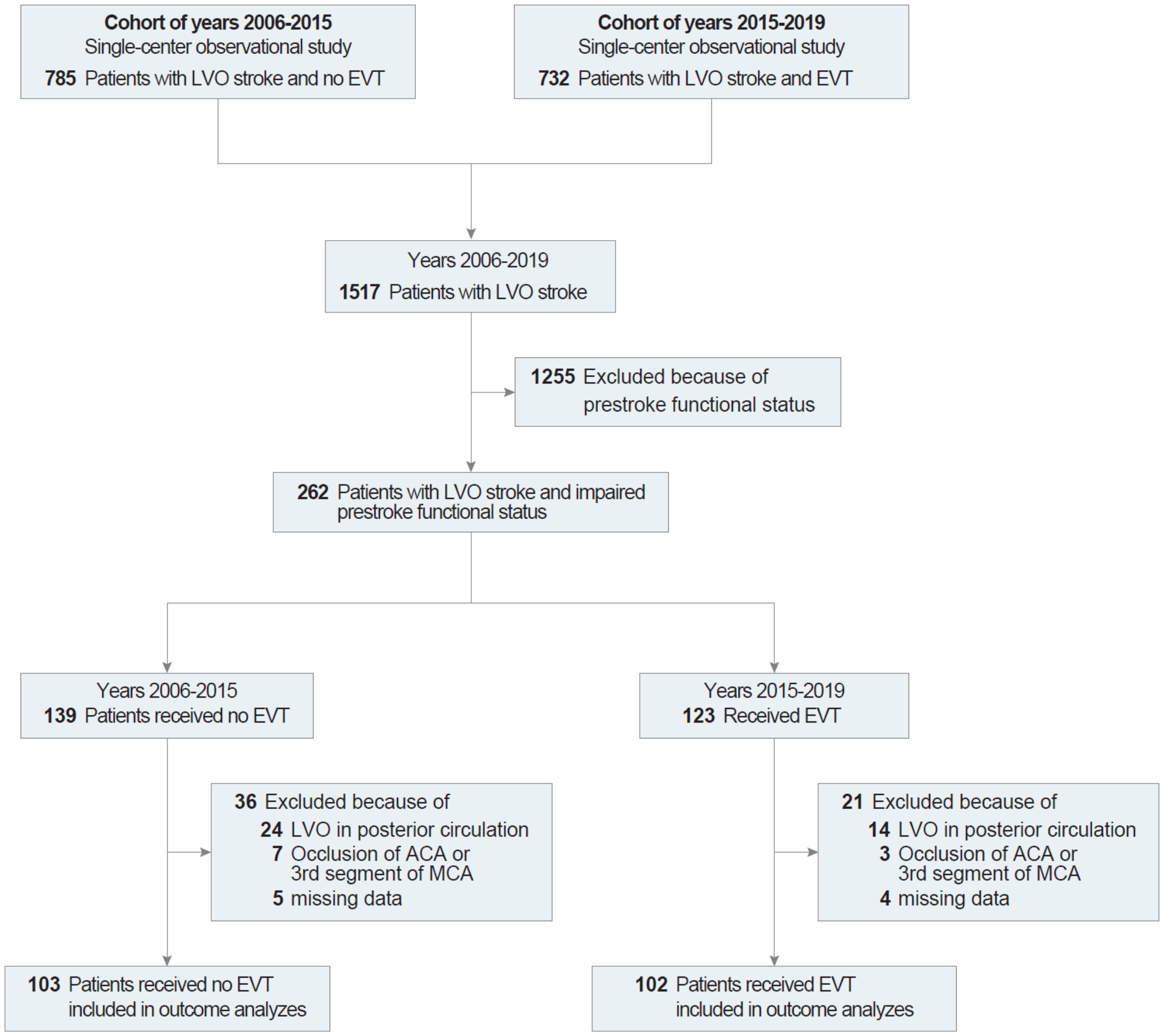

Overall, 262 patients with large vessel occlusion stroke and prestroke disability were included; 139 patients received no EVT (control group, admitted between January 1, 2006 and December 31, 2015) and 123 patients received endovascular therapy (EVT group, admitted between January 1, 2015 and June 30, 2019). After exclusion of 36 patients in the control group and 21 patients in the EVT group, 205 patients were included in outcome analyses. Abbreviations: LVO, large vessel occlusion; EVT, endovascular therapy (thrombectomy); ACA, anterior cerebral artery; MCA, middle cerebral artery.

© 2022 Sprügel MI et al. *JAMA Network Open.*

eFigure 2: Subgroup analysis of functional dependency (secondary outcome)

	Underwent Treatment No./ Total No.	Adjusted Difference between Thrombectomy and Control (95% CI)	P Value for Interaction
**All patients**	102/205	8.9 (-2.5 to 20.2)	
**Age**			
<80 years	32/74	7.2 (-13.6 to 27.9)	.62
≥80 years	70/131	9.8 (-2.9 to 22.5)	
**Sex**			
Women	74/149	6.2 (-6.1 to 18.6)	.39
Men	28/56	17.1 (-7.8 to 42.1)	
**Prestroke functional status**			
mRS=3	82/162	12.3 (-1.9 to 26.4)	.15
mRS=4	20/43	-3.1 (-24.6 to 18.5)	
**Occlusion site**			
ICA	31/75	13.3 (-5.4 to 32.0)	.68
M1	57/103	2.1 (-14.1 to 18.2)	
M2	14/27	15.7 (-20.6 to 52.1)	
**Type of stroke onset**			
Unwitnessed	51/113	5.2 (-8.5 to 18.8)	.99
Witnessed	51/92	11.5 (-6.7 to 29.7)	
**Non-contrast imaging**			
ASPECTS>5, NIHSS>5 (ESCAPE trial)			
yes	83/146	9.9 (-4.8 to 24.7)	.03
no	19/59	-4.5 (-15.9 to 6.8)	
ASPECTS>5, NIHSS>7 (RESILIENT trial)			
yes	77/135	11.7 (-3.3 to 26.8)	.02
no	25/70	-3.6 (-16.6 to 9.4)	
ASPECTS>6, NIHSS>5 (REVASCAT trial)			
yes	76/129	7.2 (-8.7 to 23.0)	.29
no	26/76	2.7 (-12.9 to 18.3)	
ASPECTS>6, NIHSS>7 (POSITIVE trial)			
yes	70/118	8.9 (-7.4 to 25.1)	.29
no	32/87	2.3 (-13.0 to 17.5)	
**Multimodal imaging**			
Clinical core mismatch (DAWN trial)			
yes	36/46	17.6 (-12.2 to 47.4)	.04
no	41/101	1.0 (-12.1 to 14.2)	
Perfusion mismatch (Defuse-3 trial)			
yes	61/95	7.3 (-11.7 to 26.4)	1.00
no	16/52	0.0 (-8.8 to 8.8)	

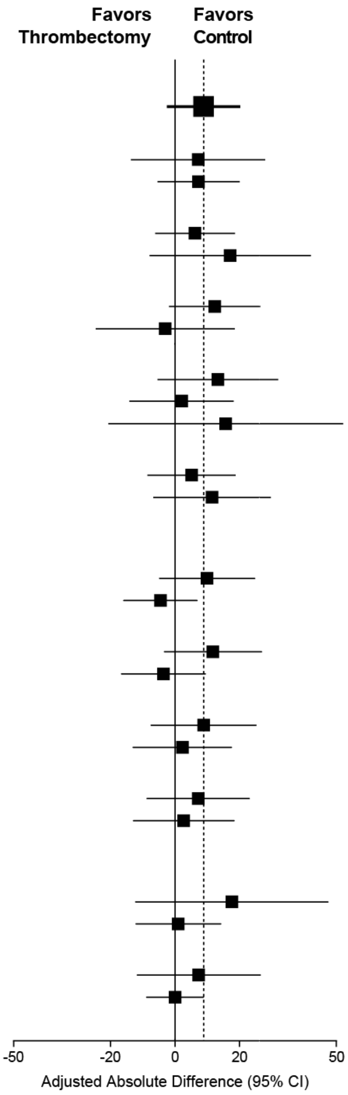

Subgroup analyses were performed using general linear model with adjustment for age, NIHSS score and occlusion site to estimate adjusted differences. ASPECTS indicates Alberta Stroke Program Early CT Score; mRS, modified Rankin Scale; ICA, internal carotid artery; M1, first segment of middle cerebral artery (MCA); M2, second segment of MCA; NIHSS, National Institutes of Health Stroke Scale.

© 2022 Sprügel MI et al. *JAMA Network Open.*

eTable: Sensitivity analyses

Study cohort	Analysis, included parameters	EVT group	Control group	Adjusted difference (95% CI)	Adjusted Odds Ratio (95% CI)	P value
Patients treated on stroke unit and/or neurointensive care unit	Multivariable regression model: Age, NIHSS score and occlusion site	20/101 (19.8%)	7/86 (8.1%)	10.3% (2.6% to 18.1%)	4.50 (1.55-13.10)	.006
Patients receiving inpatient rehabilitation	Multivariable regression model: Age, NIHSS score and occlusion site	10/48 (20.8%)	6/36 (16.7%)	13.6% (-2.6% to 29.9)	3.32 (0.78-14.19)	.10
Patients directly admitted to the University Hospital Erlangen	Multivariable regression model: Age, NIHSS score and occlusion site	14/68 (20.6%)	4/69 (5.8%)	11.0% (0.8% to 21.2%)	3.72 (1.09-12.71)	.04
Patients with available data on perfusion lesion volume on admission	Multivariable regression model: Age, NIHSS score, occlusion site and volume of perfusion lesion	14/77 (18.2%)	6/70 (8.6%)	7.7% (0.3% to 15.0%)	4.75 (1.24-18.16)	.02
Patients with large vessel occlusion stroke and prestroke disability (full study cohort)	Inverse probability treat-ment weighting: Atrial fibrillation, anticoagula-tion, hypertension, previ-ous ischemic stroke or TIA, ICA occlusion, first segment of MCA occlusion	20/102 (19.6%)	8/103 (7.8%)	12.5% (2.9% to 22.1%)	3.04 (1.25-7.36)	.01

Sensitivity analyses were conducted for the primary outcome (functional recovery at 90 days). The first was confined to patients treated on stroke unit and/or neurointensive care unit to account for the level of stroke treatment provided. Further sensitivity analyses were confined to

© 2022 Sprügel MI et al. *JAMA Network Open.*

patients receiving inpatient rehabilitation and to patients directly admitted to the University Hospital Erlangen (excluding those patients transferred from other stroke centers). The fourth was a multivariable regression model that included the initial volume of perfusion lesion. The last sensitivity analysis was an inverse probability treatment weighting (IPTW) analysis adjusted for parameters showing differences in intergroup comparison (standardized mean differences >0.10).

© 2022 Sprügel MI et al. *JAMA Network Open.*
